# Gene drives for schistosomiasis transmission control

**DOI:** 10.1371/journal.pntd.0007833

**Published:** 2019-12-19

**Authors:** Theresa Maier, Nicolas James Wheeler, Erica K. O. Namigai, Josh Tycko, Richard Ernest Grewelle, Yimtubezinash Woldeamanuel, Katharina Klohe, Javier Perez-Saez, Susanne H. Sokolow, Giulio A. De Leo, Timothy P. Yoshino, Mostafa Zamanian, Jutta Reinhard-Rupp

**Affiliations:** 1 Department of Chemical Engineering and Biotechnology, University of Cambridge, Cambridge, United Kingdom; 2 Department of Pathobiological Sciences, School of Veterinary Medicine, University of Wisconsin-Madison, Madison, Wisconsin, United States of America; 3 Global Health Institute of Merck (KGaA), Eysins, Switzerland; 4 Department of Zoology, University of Oxford, Oxford, United Kingdom; 5 Department of Genetics, Stanford University School of Medicine, Stanford, California, United States of America; 6 Hopkins Marine Station, School of Humanities and Sciences, Stanford University, Pacific Grove, California, United States of America; 7 Department of Microbiology, Immunology & Parasitology, College of Health Sciences, Addis Ababa University, Addis Ababa, Ethiopia; 8 Global Schistosomiasis Alliance, Munich, Germany; 9 Laboratory of Ecohydrology, Ecole Polytechnique Fédérale de Lausanne, Lausanne, Switzerland; 10 Woods Institute for the Environment, Stanford University, Stanford, California, United States of America; 11 Marine Science Institute, University of California, Santa Barbara, California, United States of America; University of the District of Columbia, George Washington University School of Medicine and Health Sciences, UNITED STATES

## Abstract

Schistosomiasis is one of the most important and widespread neglected tropical diseases (NTD), with over 200 million people infected in more than 70 countries; the disease has nearly 800 million people at risk in endemic areas. Although mass drug administration is a cost-effective approach to reduce occurrence, extent, and severity of the disease, it does not provide protection to subsequent reinfection. Interventions that target the parasites’ intermediate snail hosts are a crucial part of the integrated strategy required to move toward disease elimination. The recent revolution in gene drive technology naturally leads to questions about whether gene drives could be used to efficiently spread schistosome resistance traits in a population of snails and whether gene drives have the potential to contribute to reduced disease transmission in the long run. Responsible implementation of gene drives will require solutions to complex challenges spanning multiple disciplines, from biology to policy. This Review Article presents collected perspectives from practitioners of global health, genome engineering, epidemiology, and snail/schistosome biology and outlines strategies for responsible gene drive technology development, impact measurements of gene drives for schistosomiasis control, and gene drive governance. Success in this arena is a function of many factors, including gene-editing specificity and efficiency, the level of resistance conferred by the gene drive, how fast gene drives may spread in a metapopulation over a complex landscape, ecological sustainability, social equity, and, ultimately, the reduction of infection prevalence in humans. With combined efforts from across the broad global health community, gene drives for schistosomiasis control could fortify our defenses against this devastating disease in the future.

**Key Learning Points**

- The modification of natural snail populations by means of gene drives could assist in the fight against schistosomiasis prevalence and transmission.
- Although molecular tools are available for the production of genetically modified snails through genome editing, they need to be adapted to *Biomphalaria glabrata*.
- Mathematical modeling of gene drives and disease dynamics can provide key understanding in the potential for success of gene drive–based intervention to control schistosomiasis.
- Gene drives have the potential to influence the global schistosomiasis disease burden, allowing for adjustments in future chemotherapeutic needs and alternative elimination efforts.
- Responsible ethical governance for schistosomiasis transmission control through gene drives is needed and could draw on existing frameworks for genetically modified mosquitoes and Mass Drug Administration.

**Top Five Papers**

- Abe M, Kuroda R. The development of CRISPR for a mollusc establishes the formin Lsdia1 as the long-sought gene for snail dextral/sinistral coiling. Development. 2019 May 1;146(9):dev175976.
- Dong Y, Simões ML, Marois E, Dimopoulos G. CRISPR/Cas9 -mediated gene knockout of Anopheles gambiae FREP1 suppresses malaria parasite infection. PLoS Pathog. 2018 Mar;14(3):e1006898.
- Woolhouse WEJ. On the application of mathematical models of schistosome transmission dynamics. II. control. Acta Trop. 1992 Feb; 50(3): 189–204.
- Sturrock RF. Schistosomiasis epidemiology and control: how did we get here and where should we go? Mem Inst Oswaldo Cruz. 2001;96:17–27.
- Kofler N, Collins JP, Kuzma J, Marris E, Esvelt K, Nelson MP, et al. Editing nature: Local roots of global governance. Science. 2018 Nov 2;362(6414):527–9.

## Introduction

Gene drives, or the purposeful spread of desired alleles throughout a population to control or modify populations of pests [1] or intermediate hosts for disease, are rapidly being developed in research laboratories [2–5]. Extensive literature exists about the molecular feasibility, ecological ramifications, and bioethics of such approaches [6,7], with most of the research effort focused on arthropods, in particular, mosquitoes that are vectors of important human diseases such as malaria and dengue fever, whereas gene drive application to other intermediate hosts is less widely discussed.

**Fig 1:**
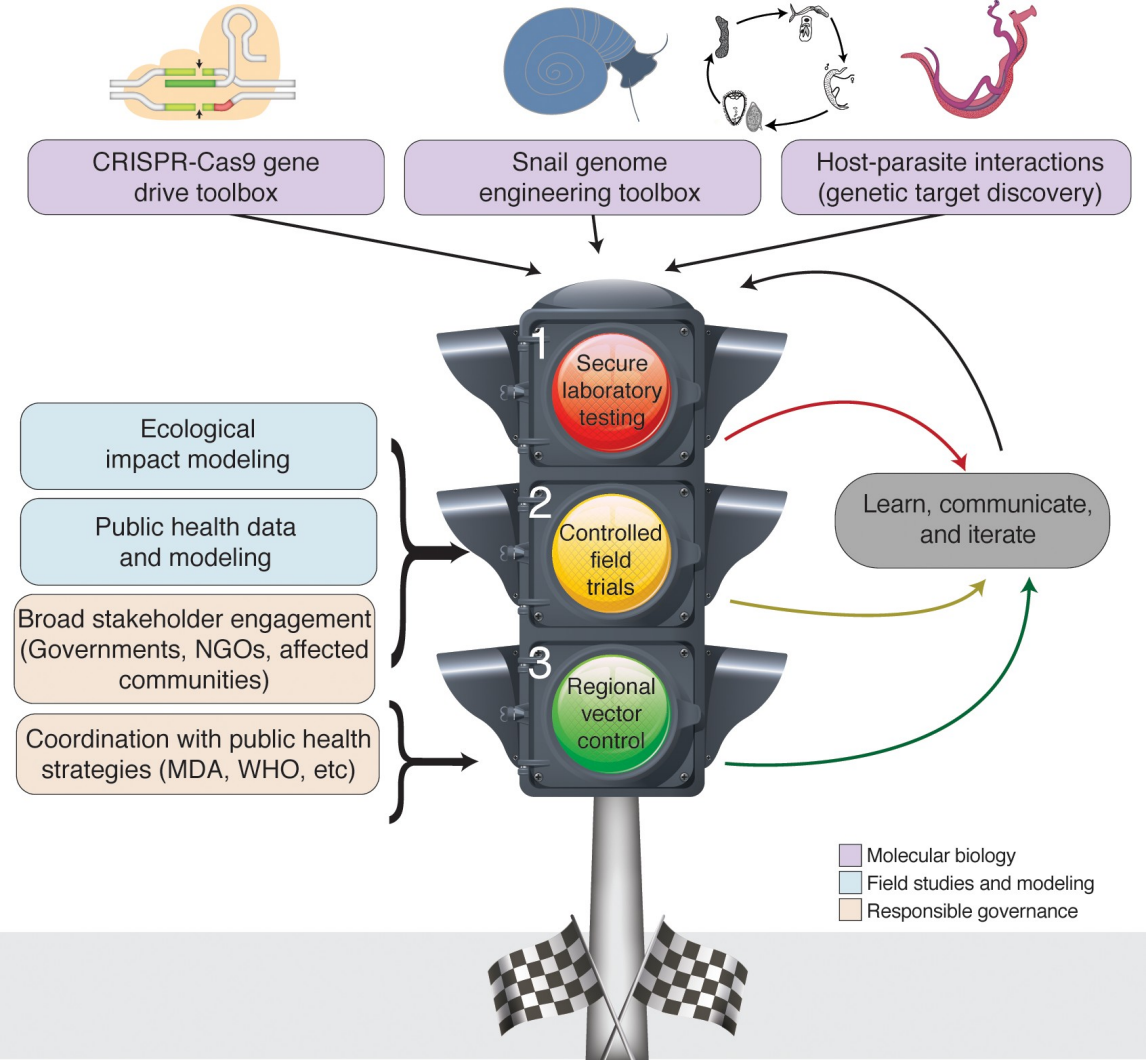
Traffic light model for the development of an antischistosome gene drive in snails. Model for the responsive technology development strategy is discussed in this review. At stage 1, research is small scale and takes place in secure laboratories. Data are communicated widely and can lead to further experimentation or, potentially, advancement to stage 2. These controlled field trials incorporate efforts from additional disciplines, including ecological impact modeling and public health, and depend on broad stakeholder engagement. As before, data are broadly disseminated, leading to iteration or potential advancement to stage 3. Regional transmission control depends on the previous alliances as well as coordination with co-occurring public health strategies such as MDA and international strategies by groups such as WHO. MDA, mass drug administration; NGO, nongovernmental organization; WHO, World Health Organization.

Mollusks, in particular, snails and slugs, can be important intermediate hosts of many parasitic worms of medical importance: they pose risk for human health and may cause relevant socioeconomic burden in the most vulnerable populations [8] living in subsistence economies and lacking access to clean water and healthcare. Mollusks can be intermediate hosts of both human diseases (such as schistosomiasis, angiostrongyliasis, opisthorchiasis, clonorchiasis, and paragonimiasis) as well as animal diseases of agricultural and economic importance (such as fascioliasis). Of these, schistosomiasis is the most predominant snail-borne disease, with 779 million people at risk for infection and 207 million individuals in 74 countries being infected [9]. Mollusks could be amenable to population control or modification through gene drives but require tailored molecular and ecological approaches; this is a result of their substantial biological differences compared with arthropods [10], to name a few: (1) many mollusks are simultaneous hermaphrodites that can self-fertilize in the wild and exhibit wide species-specific variations in reproductive preferences and generation times [11], (2) mollusks may inhabit both terrestrial or aquatic ecosystems, (3) some mollusks can aestivate (a type of hibernation) in times of stress [12], and (4) some parasites can cause parasitic castration in mollusks [13]. These differences will cause gene drives targeted at different aspects of mollusk biology (e.g., reproduction, resistance) to behave in a potentially different way than those promulgated in arthropods. In this review, we discuss the necessary molecular, modeling, and regulatory steps to determine the potential relevance and impact of gene drives, focusing on the most widely distributed and well-studied of snail vectors, *Biomphalaria glabrata*, a host of *Schistosoma mansoni*, as a gene drive model (Fig 1). We call for further input from the community and provide a foundation upon which to continue the discussion. We provide supportive evidence suggesting that the development of gene drives for *B*. *glabrata* could be technologically feasible in the near future and that gene drives could provide untapped opportunities for the control of the intermediate host of schistosomiasis. We emphasize that more data and models are required to make evidence-based predictions of the context-specific outcomes of such gene drives and that structured stakeholder engagement, starting now, will be key to guide the responsible development of this potentially transformative tool for schistosomiasis transmission control.

### The snail intermediate hosts of schistosome parasites

Freshwater snails serve as obligate intermediate hosts in the life cycle of all schistosome species, within which asexual reproduction of intramolluscan developmental stages of the parasite results in a drastic expansion of larval schistosome populations. The snail-infective parasitic stage, the miracidium, hatches in the environment from an egg passed in human stool or urine and must find a suitable snail host within hours to begin its development within snail tissues [14]. Through an asexual reproductive process, a single miracidium entering a snail transforms into a mother sporocyst that is capable of generating many successive, intramolluscan larval stages, culminating in the production and release of hundreds to thousands of free-swimming human-infective cercariae per day into the water. *B*. *glabrata* is one of the primary models used to study these intermediate hosts. *Biomphalaria* spp. are native to the neotropics and become sexually mature at approximately 4 to 6 weeks of age. They are freshwater animals that have adapted to a range of conditions, such as flowing and standing water, where they can be found at a variety of depths and from tropical to arid environments [15,16]. Unsuitable habitats include marine environments, salt marshes, or locations with fast-flowing water [17,18]. Snails can survive drought over a sustained period of time with differences across species: for example, in areas with an annual dry season, *Biomphalaria* spp. snails have been known to survive for 5 to 7 months under mud or other sheltered areas by aestivating [15], thanks to adaptive regulation of their metabolic and respiratory activities [12,15,19]. The *B*. *glabrata* genome has been sequenced and provides a critical resource to better study its biology, especially components that contribute to snail immunity, and enables new technologies to pave the way for genome editing and gene drives [20]. Although *B*. *glabrata* is a known model species to study schistosomiasis, it is important to note that there are distinct species in the snail phylogeny that serve as intermediate hosts. This means it is likely not possible to apply a method to all snails based on a single species. There are 4 snail genera that comprise the majority of intermediate host species of schistosomes worldwide: *Biomphalaria*, *Bulinus*, *Oncomelania*, and *Neotricula* [14]. *B*. *glabrata* is a snail in the gastropod subclass Heterobranchia and family Planorbidae [21] and provides a model for the many *Biomphalaria* species that transmit *S*. *mansoni*. *B*. *glabrata* can likely also be a model for *Bulinus* spp., which are also planorbids. It is less likely that *B*. *glabrata* will inform relevant methods of *Oncomelania* and *Neotricula* snails that belong to a different gastropod subclass, Caenogastropoda [21]. In short, additional research efforts will be needed to adapt relevant methods for these distinct types of snails.

Most snail–parasite relationships are highly specific, especially those involving schistosomes, and the variety in taxonomic compatibility complicates control efforts [22]. For example, some snails can host *S*. *haematobium* or *S*. *bovis* from regions in South, West, and East Africa, whereas these snail species cannot host the same schistosome species found in regions of Egypt and the Middle East [23,24]. Schistosomiasis is not exclusively limited to tropical countries in Africa, Asia, and South America [25–27], but it has also been found in Europe, with hybrid schistosomes causing urogenital schistosomiasis hosted in snails with habitats in France, Germany, and Italy [28–30], thereby introducing a new array of climate-related considerations [31].

## Past and current methods of intermediate host control

In the past, a variety of approaches for snail control has been used, including infrastructural, chemical, and biological interventions [25]. Japan was the first country to completely eliminate schistosomiasis thanks to a comprehensive, multifaceted strategy: at first, snails were found and killed by hot water and flamethrowers (a technique later replaced by the use of molluscicides); the land was modified to minimize exposure of humans to snail-rich areas (e.g., through cementing water canals in agricultural fields); hygiene education served to minimize water contamination by feces; and irrigation canals were cemented to reduce the availability of water sources that served as good snail habitat [32]. The mechanization of agriculture allowed Japan to reduce reliance on oxen as a production animal, thus reducing the abundance of oxen as alternative definitive hosts of *S*. *japanicum* [33]. Other physical methods, such as controlling natural water flow, have been used to decrease schistosomiasis transmission (comprehensively reviewed elsewhere [34]). Reported success stories elicit an important lesson: only through a combination of different strategies, e.g., biomedical interventions combined with environmental strategies to interrupt transmission, such as molluscicide use or water, sanitation, hygiene (WASH) initiatives [35], has localized elimination been achieved. Interestingly, a recent study analyzed the effectiveness of using vector control or the antischistosomal drug praziquantel, or both, on reducing schistosomiasis prevalence. After conducting a global assessment of these methods and their combination, it emerged that snail control, alone or in combination with other strategies, proved to be the most effective in reducing schistosomiasis prevalence [25].

One of the most widely used techniques for snail control has been the application of molluscicides, with the most common chemical molluscicide being niclosamide [36]. Molluscicides are compounds that are toxic to snails and that have been shown to reduce snail populations when applied in bodies of water or on muddy surfaces [37–39]. Although effective, molluscicides are associated with significant drawbacks: their effects are often not strictly specific to snails, and because of their impact on animals such as fish and amphibians, they require specific dosage calculations to minimize off-target effects [40]. Other drawbacks to using molluscicides include their lack of residual killing effect, which results in the inevitable recolonization by snails and the need for multiple applications that increase the cost of deployment [10,22,41]. There is evidence for unintended knock-on effects, such as the slower decomposition of snail cadavers by flies when snails have been treated with molluscicides [42]. Alternatives to synthetic chemical molluscicides are natural snail-killing compounds such as salts, latex, or plant-derived saponins [43–46], which can alleviate the high cost associated with chemical molluscicides by being locally sourced.

Chemical methods for control can be effective, but the potential detrimental effects on the environment and their high cost can make them an unattractive option [34,47]. Biological methods for the control of the intermediate snail hosts, including the use of snail competitors, bacterial pathogens of snails, or predators such as fish or prawns, provide a potential alternative [48–51]. However, these methods may also have unintended off-target effects on local fauna and flora, especially if exotic agents are introduced [48–51]. For example, a potential biological control method using cyanobacteria as a molluscicide has been tested but was toxic to nonvector snail species [52]. Other biological control methods have risks, such as using a highly invasive snail where replacement of a native species [48,49] may have unintended consequences on the ecosystem. In Senegal, a native, freshwater migratory prawn (*Macrobrachium vollenhoveni*) was reintroduced after its unintended extirpation following the construction of the Diama dam to control snail populations through predation in experimental settings and resulted in both a significant reduction of the infected snail population and schistosomiasis prevalence in the area [53]. However, artificially maintaining high abundances of biological control agents to effectively reduce snail density often presents formidable challenges, especially if scaled up to wide geographical areas, and the risks of negatively impacting the natural environment may sometimes outweigh the benefits of employing biological control methods. Two things are abundantly clear from the previous review of snail control strategies: (1) reducing snail populations can have a direct and positive impact on local human infection prevalence (and presumably intensity), and (2) currently available snail control approaches are not sustainable in the long term.

## Gene drives for intermediate host control

In view of the shortcomings of snail control methods outlined previously, new approaches to interrupting schistosome transmission at the intermediate host stage are needed. With recent technological advances in genome editing, it may now be possible to genetically modify natural snail populations using gene drives. Intermediate host control via gene drives relies on the super-mendelian inheritance of engineered traits in sexual reproduction (reviewed elsewhere [54]) and is broadly divided into 2 main categories: population suppression and population replacement. Population suppression drives are designed to crash a population and can utilize a number of different strategies such as the Maternal effect dominant embryonic arrest (*Medea*) system [1], a driving endonuclease gene (DEG) that results in sterility [2,3], or a DEG that causes sex-biasing in reproduction [55,56]. Population replacement drives, on the other hand, can also use a toxin–antidote design [57] or DEGs to drive resistance alleles throughout a population [4,58]. If the DEGs or driven alleles do not confer too large of a fitness cost, they can eventually be driven to fixation in the population, effectively replacing the wild-type population with a resistant population.

### Population suppression drives

Population suppression drives are the most advanced for the control of arthropod intermediate hosts that transmit parasites like *Plasmodium* spp., the protozoa responsible for malaria, but face particular challenges when being applied toward schistosomiasis control. Schistosome intermediate hosts of the genera *Biomphalaria* and *Bulinus*, which are simultaneous hermaphrodites that can self-fertilize in the wild [59], would thus be refractory to gene drive sex-biasing but would be amenable to alternative gene drive approaches for population suppression; on the other hand, *Oncomelania* spp. snails are dioicious and would be amenable to all of the aforementioned. Simultaneous hermaphroditism could thwart sex-biasing drives, so population suppression through a *Medea* system or an underdominant genetic load drive is better suited for schistosome intermediate hosts, as these approaches result in the death or decreased fitness of heterozygotes and enable the survival of homozygotes, which continue to pass on the driven element through self-fertilization or outcrossing [60,61]. Population suppression drives rely on the specific targeting of genes that are essential for reproduction or development, and thus, their implementation would require the identification and validation of such genes in schistosome intermediate hosts. These targets could be discovered via reverse genetic screens, which have been successful in other model and nonmodel organisms. RNA interference (RNAi) has been successfully used in *B*. *glabrata* [62,63], but to identify genes essential in development, this technique needs to be adapted for embryonic and egg-stage snails.

### Population replacement drives

**Fig 2:**
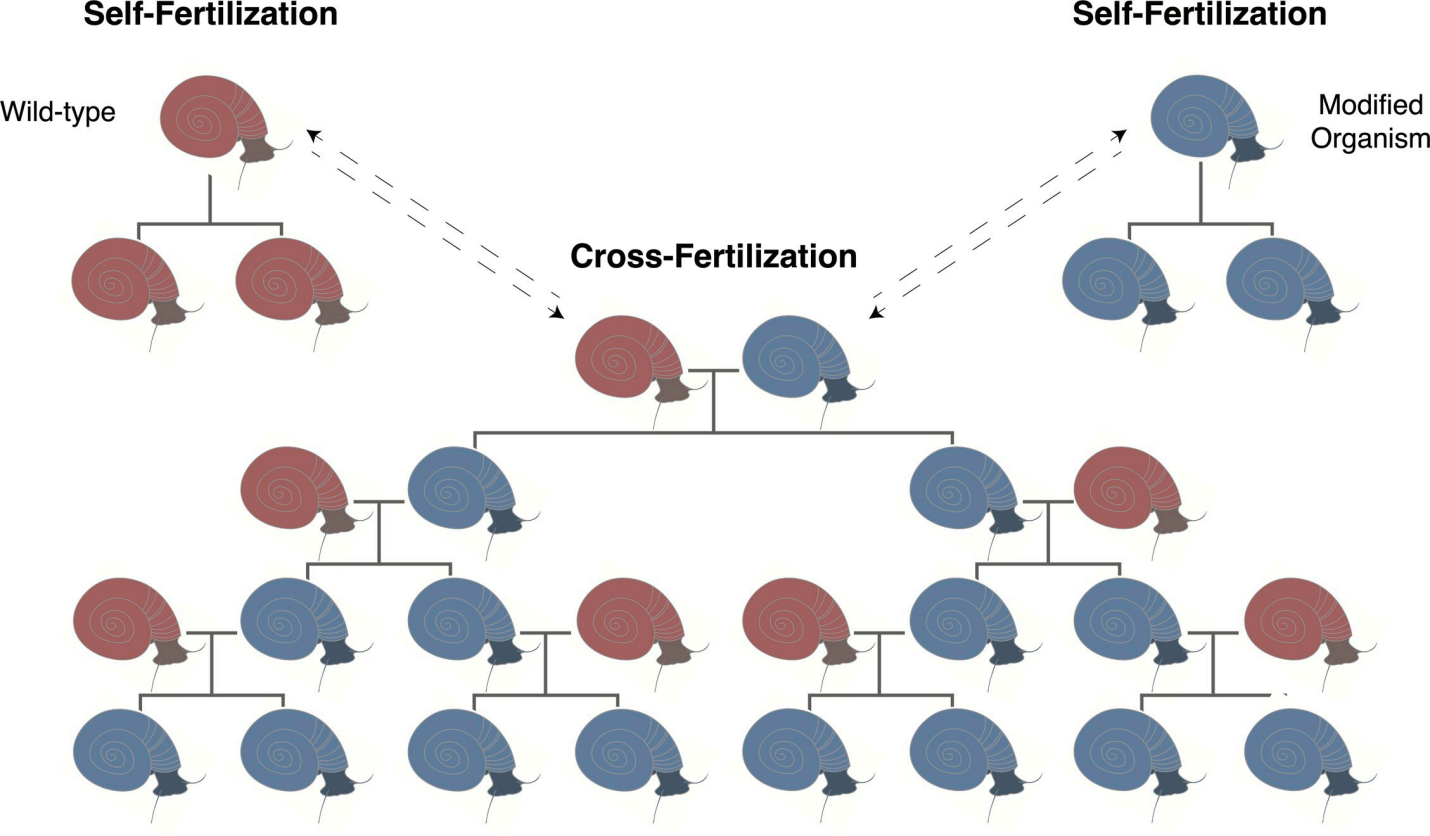
Snail gene drive schematic. A gene drive with the goal of replacing wild populations could be modeled similarly to drives under development in arthropods—given a high enough transmission rate and a low enough fitness cost, a trait can eventually be driven to fixation. However, in hermaphroditic snails like *Biomphalaria glabrata* and other schistosome-transmitting intermediate hosts, the ability to self-fertilize needs to be taken into account. Most schistosome-transmitting snails can perform both self- and cross-fertilization, but preferences by distinct species or strains have been observed, and these will need to be considered in future modeling and implementation efforts. Potential cross-fertilization between 2 wild-type and 2 modified snails is not shown.

In contrast, population replacement drives (Fig 2) for schistosomiasis control can build on a broader base of preexisting knowledge. *B*. *glabrata* boasts a rich history as a model for invertebrate immunology, and much is known about the genetic basis of host–parasite compatibility in this system [64,65]. The availability of strains that differ in their susceptibility to schistosome infection [65–67] could provide leads for a population replacement strategy, and work is underway to identify loci that may confer schistosome resistance in lab-derived and wild isolates of *B*. *glabrata* [68–72]. Though host–parasite compatibility is a complex phenotype, there are several genes already known to be involved in pathogen recognition and/or associated with parasite resistance, including fibrinogen-related proteins [63,73–75], thioester-containing proteins [76], and Toll-like receptors [77,78], and enzymes involved in parasite killing mediated by reactive oxygen species [68,79–84]. Recently, a genomic region, the Guadeloupe Resistance Complex (GRC), has been shown to contain alleles that are associated with resistance in experimentally evolved lines of *B*. *glabrata* [72]. The GRC is a <1 Mb region that contains a dominant allele that confers an 8-fold decrease in infectivity. In total, this region contains 15 coding genes, including 7 transmembrane proteins with possible roles in parasite recognition, which could be further characterized to identify the protective mechanism and relevant gene (or genes) involved. As suggested by the discoverers of the GRC, this dominant allele could be coupled to a CRISPR-mediated gene drive and spread through wild snail populations in order to confer resistance to parasitic infection [72].

Beyond naturally occurring alleles as potential candidates for a population replacement drive, one can imagine the engineered expression of synthetic elements, such as parasite–toxic miRNAs or neuropeptides, to help fight off infection and/or prevent development of the human-infective cercarial stage. RNAi screens of pertinent intramolluscan schistosome stages could reveal essential genes for parasite persistence and development, and targeting these via the transgenic expression of siRNAs could be a parasite control strategy [85].

Overexpression or knockout of one or a number of these candidates may bolster snail defense to schistosomes and, ultimately, improve public health. New data from genetic approaches are improving our molecular-level understanding of snail–parasite interactions, but it remains difficult to link resistance traits to single genes. Arguably, the precise mechanism of a given resistance allele need not necessarily be fully understood in order to be efficacious in engineered snails. However, available knowledge of off-target or epistatic effects of an engineered allele must be taken into consideration during strain engineering, and improved molecular characterization of these candidate genes will only increase the likelihood of their safe and efficacious application in population replacement gene drives.

Importantly, replacement drives decrease the intermediate host’s parasitic load without crashing the snail population and therefore may be expected to largely avoid ecological consequences on, for example, the snail’s predators or the forage base.

The introduction of population replacement strategies requires careful analysis of the associated fitness costs that parasite resistance might have on gene drive–carrying populations. For instance, artificially selected resistant *B*. *glabrata* have been shown to exhibit reduced fertility, regardless of parasite infection status [86], whereas infected snails exhibit castration or reduced fecundity after parasite infection [87,88]. Assessing the interplay of these traits and their consequence on the fitness of snails with the driven allele is therefore key to comprehensively evaluating the feasibility of population replacement gene drives for schistosome intermediate hosts (see “Impact Modeling”).

### Developing the snail genome engineering toolbox

The previous discussion remains theoretical as long as the necessary molecular tools to develop these types of gene drives, such as germline transgenesis and active DEGs, are not adapted to schistosome intermediate hosts. CRISPR-Cas9 could be adapted in *B*. *glabrata*, as it has been used in the related mollusks *Crepidula fornicata* [89] and a trematode vector *Lymnea stagnalis* [90]. Germline transgenesis has also recently been achieved in the bivalve mollusk, *Crassotrea gigas*, through the piggyBac transposon system and sperm-mediated gene transfer [91]. Concurrently, promoters that are active in the germline will need to be identified and used to drive expression of the gene drive components such as Cas9 and its guide RNA (gRNA). One advantage in using *B*. *glabrata* as a model for this work is the availability of the only Lophotrochozoa (the superphylum that houses gastropod mollusks) immortalized cell line, the *B*. *glabrata* embryonic (Bge) cell line. Although this cell line was established nearly 50 years ago (and by this point is highly diverged from *B*. *glabrata* [92–94]), it may prove useful to study the determinants of CRISPR genome editing outcomes in snail cells, including the mechanisms of snail DNA repair pathways. If CRISPR-Cas9 is the DEG technique of choice, the cell line will also aid in identifying and validating CRISPR gRNAs and in optimizing the parameters for the endogenous homology-directed repair process that copies the driven gene to a homologous chromosome. Although it has been rarely utilized, transgenesis has been described in this cell line [95–97], allowing for screening of promoters and gRNAs. Once the snail genome engineering toolbox has been developed, there will be challenges to translate this to the field, including, but not limited to, environmental safety and biosafety assessments, regulatory requirements, and strong public support (addressed here in the section “Stakeholder considerations and ethical implications” [98,99]).

## Modeling the potential epidemiological impact

A population-modifying gene drive has the potential to reduce or (when used concurrently with existing treatments) eliminate schistosomiasis locally. However, knowledge on its potential efficacy is limited. Further understanding is required as to how introduction could change the local genetic landscape for snail species and whether possible negative changes could be outweighed by expected improvements in human health. In this context, as in many others, mathematical modeling can be used to explore the utility of this new strategy in a system with complex behaviors and thereby provide the scientific basis for ethical and political considerations.

Key to determining the success of any intermediate host control strategy is finding a reliable metric for human health. A typical means to quantify disease burden and a change thereof has been the concept of disability-adjusted life years (DALYS) [100]. Disability is an important concern for the NTD community, including those affected by or working to reduce schistosomiasis. However, the disagreements and controversies about the correct DALYs to be attributed to schistosomiasis over the recent years since the Global Burden of Disease Study in 2010 indicates just how difficult it is to quantify disease impact [101,102]. Estimates of the global burden of schistosomiasis have thus ranged from 1.7 million DALYs to as many as 56 million DALYs, depending on the disease prevalence levels applied and the morbidities (and their associated disability estimates) included in the calculations [103]. Furthermore, because many regions endemic for schistosomiasis are also coendemic with other diseases, it is difficult to attribute exact causes of disability and the downstream socioeconomic impact [100,101]. Most importantly, DALYs are based on prevalence and not intensity of infection, whereas pathology in schistosomiasis is invariably associated with the rate of egg production, a function of the number of mated worms. When assessing the impact of gene drive–based control, it will be thus preferable to use reduction in infection intensity as measured by egg count in urine and stool [104,105]. Further indications that could help to demonstrate the impact of gene drive–based control include reinfection rates in populations that have previously undergone treatment, as well as infection rates in children not previously infected. Fewer new infections and reduced infection rates would suggest that gene drive–based control was indeed successful in interrupting the schistosome lifecycle. Moreover, depending on the gene drive strategy employed, either a reduction in cercaria-shedding snails or a reduction in snail prevalence will also indicate success of the intervention.

More research looking into how different levels of infection intensity translate into morbidity, including stunting, anemia, malnutrition, and reduced cognitive and work performance is essential [106]. Measurement of nonhealth effects, such as fatigue and school attendance, can also serve to evaluate long-term success of morbidity control activities [107–109].

**Fig 3:**
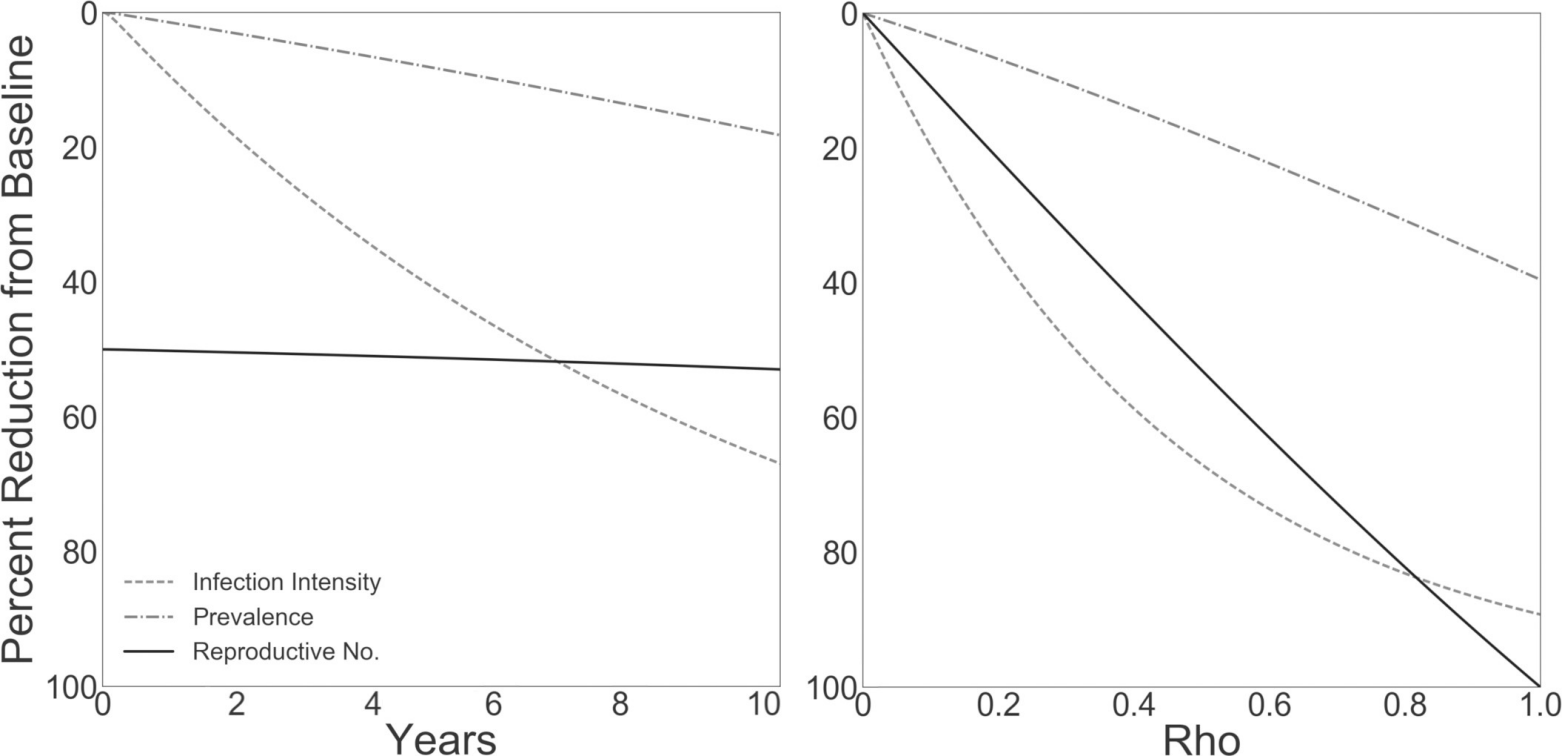
Simulated impact of engineered snail release on worm burden in humans. Reduction from endemic equilibrium in infection intensity (as measured by per human egg shedding rate, approximately, *w_t_*), prevalence of infection in humans (*Ω_t_*), and effective reproductive number (*R_t_*) of the schistosomes. (A) Percent reduction over 10 years when engineered snails are maintained at 50% frequency (*ρ* = 0.5) in the population. (B) Percent reduction after maintaining engineered snails at frequency *ρ* for 10 years.

In contrast to the theoretical and empirical foundations that exist to explain schistosome disease dynamics, there is no clear understanding of the dynamics of gene drives in a wild population. A few recent studies have modeled general gene drive behavior [110,111], but the need remains to understand gene drive behavior and impact in schistosomiasis before implementing it as a tool to control the disease. Here, we use the classic framework presented by MacDonald [112] and reiterated by Woolhouse [113], adding a single term (ρ = proportion of snails resistant to infection) to include the effect of engineered resistance to infection in snails on schistosomiasis transmission dynamics (see S1 Appendix). We use this model to illustrate the qualitative behavior of transmission patterns as a result of a successful introduction of engineered resistant snails. In Fig 3, we show how transmission and adult worm burden (prevalence and intensity) vary over a 10-year time period of moderately successful introduction and how transmission and adult worm burden vary with introduction success after 10 years.

This simple simulation provides us with a qualitative understanding of how, on average, successful introduction of these snails may reduce schistosomiasis in endemic regions. When transmission rates are reduced, humans with the most intense infections could experience the most dramatic percent reduction in infection intensity because of the natural mortality of adult worms. This feature, along with the uneven distribution of adult worms in the human population, explains why the reduction in prevalence of schistosomiasis in humans occurs more slowly than the reduction in intensity of infection. Hypothetically, the disease will be locally eliminated when all adult worms have perished if the engineered snails maintain 100% efficiency in removing potential intermediate hosts from the environment. Because the longevity of the adult worms can be several years to decades (see S1 Appendix), parasite reduction proceeds slowly with snail or environmental intervention alone and will occur on the order illustrated in Fig 3. Current anthelmintic treatment is necessary for faster elimination. Under reasonable conditions, given this framework and its associated assumptions, successful implementation of engineered snails could have measurable impact in endemic regions. The concavity of the curves in Fig 3 illustrates that reduction in prevalence and intensity of infection occurs at low frequencies of engineered snails, with diminishing returns at higher frequencies. This behavior indicates that achieving high frequencies of the engineered variant may not be necessary to see measurable reduction in disease burden.

Panel A in Fig 3 illustrates a scenario in which drive introduction is 50% efficient in removing potential intermediate hosts from the environment. Inefficiency can occur either by failure of the drive construct to reach 100% frequency or by failure of the construct to promote 100% resistance to infection in snails. Several factors have the potential to inhibit success. For example, unlike mosquito manipulation in malaria control efforts, gene drives in schistosomiasis control will be challenged by the ability of the snail host species to self-fertilize. It is not clear how the tendency to self-fertilize in these snails will affect the ability of an engineered variant to establish in a natural population and have meaningful impact on disease transmission. Likewise, with population replacement approaches, the success of an engineered variant may depend on the occurrence of natural resistance in the local snail population. Snail population sizes in many schistosomiasis-endemic areas are subject to natural fluctuations according to aquatic habitat availability that changes with precipitation, hydrological dynamics [114], and the physicochemical characteristics therein [115]. Incorporation of realistic spatial and temporal population dynamics is essential for a model to give accurate predictions of the frequency changes of engineered variants and of schistosome transmission rates between snails and humans. Finally, because snail vectors of schistosomes also serve as intermediate hosts of other trematode species, frequently in coinfections [116], the influences of such interspecific trematode interactions on schistosome survival may not be predictable in genetically modified snails. These considerations illustrate the need for comprehensive modeling and empirical tests to inform any efforts to introduce engineered snails in endemic areas [117].

Currently, ongoing activities that are part of the morbidity control and elimination strategies as outlined by the World Health Organization (WHO) are preventive chemotherapy by means of mass drug administration (MDA), WASH activities, hygiene education, and snail control [118]. Understanding how the dynamics and pace of disease reduction can be influenced by the additional control measure of gene drives would be important to better forecast future chemotherapeutic needs and duration of elimination activities. The converse is also true: it is imperative to account for ongoing control activities to inform gene drive–based control efforts. For example, ongoing control efforts can influence immunity of the human host, habitat suitability for snails, or schistosome–snail infection dynamics [119–128], which should be included in models. These models can predict meaningful impact by comparing reduction in morbidity and schistosomiasis prevalence with and without gene drive intervention. These models can also elucidate interactions and outcomes of a multifactorial schistosomiasis elimination strategy that potentially includes gene drives as a new tool [129].

To enable the implementation of gene drives in schistosomiasis intermediate host control, many technological advances are required. However, such scientific progress cannot be viewed in isolation and requires researchers, policy makers, and other stakeholders to also consider ecological and ethical implications.

## Stakeholder considerations and ethical implications

Gene drives in wild populations have raised significant ethical challenges [130]. The National Academies of Science, Engineering, and Medicine (NASEM) have emphasized the importance of an interdisciplinary perspective on gene drive research that explicitly attends to complex human values and the necessity of the community, stakeholders, and public engagement to accompany technical research and development [130]. Although decision-making involves risk assessment, the prevailing uncertainties of genome engineering technology in snails and other organisms and its behavior in the wild impede accurate risk/benefit analysis [131]. Therefore, some have emphasized the need to allow for sufficient time to develop amendments to current regulatory frameworks [132].

While research is ongoing, and governance structures take shape, a thoughtful engagement plan should be included that considers relevant communities, stakeholders, and the global public throughout the process, from early research and development through—if applicable—the release and monitoring of modified organisms in the environment. Target Malaria, a not-for-profit organization currently developing a gene drive to control malaria-transmitting mosquitoes, provides one example, with a dedicated stakeholder engagement team at each of its African locations: Burkina Faso, Mali, and Uganda [133]. These teams engage stakeholders at all levels, from the local villages where entomological collections are performed to the international level. However, although such engagement efforts have been welcomed by many stakeholders, they have not been without controversy: accusations of politicization of the process and fears of unintended effects by local stakeholders serve as a warning against technocratic solutions [134,135]. Thus, such challenges and fears should be carefully weighed by gene drive researchers and supporters, and public opinion should be monitored and valued throughout the process.

Using these precedents, the governance and implementation of public engagement for schistosomiasis transmission control through a gene drive might be structured in one of the following ways: MDA-based governance, mosquito control–based governance, and hybrid model.

### MDA-based governance

Partnerships could be integrated with the governance system that is in place for MDA and existing disease control programs. Field research with gene drives will involve Institutional Review Boards (IRBs) and Ministries of Health, whose responsibilities are to foster advancement with dedicated technical working groups. Engagement of the local community could be achieved through existing groups that distribute MDA and encourage WASH compliance.

### Mosquito control–based governance

Alternatively, the guidance framework for the testing of genetically modified mosquitoes developed by WHO’s Training in Tropical Diseases (WHO-TDR) or the lessons gleaned from other efforts in releasing modified mosquitoes could be adapted for testing and releasing genetically modified snails [136,137]. WHO-TDR advises that the ethics and engagement components of a genetically modified mosquito research program take place at multiple levels of the study’s trial and regulation, emphasizing the need to have all levels addressed concurrently (Fig 1). The framework further outlines that (1) each country has its own sovereign regulatory process, but overarching international agreements or treaties may also be relevant, and (2) early interaction with regulators is advised in order to identify the appropriate regulatory pathway.

### Hybrid model

An alternative approach is the use of a hybrid model that draws upon a combination of current MDA governance and newer models established for the regulation of research and release of genetically modified mosquitoes. This hybrid model would allow for coordinated overlap between MDA and snail study/release where appropriate (so as to not “reinvent the wheel”) but would also contain structures uniquely tailored for the release of modified snails and the epidemiological context of schistosomiasis transmission. These might include a national-level body that oversees the technical review of the proposed study or trial, a collaborative academic institution that contributes expertise and provides regulatory structures such as IRBs and Institutional Biosafety Commissions (IBCs), and regionally guided mechanisms for community engagement. In addition, there will also be a need for rigorous site selection criteria to ensure appropriate local oversight of the study. Though perhaps less of a possibility with snails than with mosquitoes, geographic boundaries might be crossed by modified organisms; therefore, an overarching international governance framework will likely be required for countries to adopt this disease control strategy, cognizant that each has its own sovereign regulatory process. With regard to the latter, some have proposed a neutral third-party coordinating body whose task would be to establish, facilitate, and report on inclusive deliberations between all involved parties, with a particular emphasis on the local impacted communities that will first be affected [138].

## Conclusions

Introduction of gene drives in snails for schistosomiasis transmission control will be complex but could be feasible in the near future. The time for a community-wide discussion of its potential impact is now. Although MDA, WASH initiatives, and community education have been instrumental in reducing the burden of disease of schistosomiasis, intermediate host control remains an essential component of the integrated disease control strategy, and cutting-edge genetic control techniques should be included as a potential addition to the portfolio for intermediate host control.

Because of its ecological specificities, the relevance of gene drives for the snail–schistosome system remains to be fully investigated. Building upon the brief introduction here, more comprehensive mathematical modeling of the potential impact of a snail gene drive should be performed, and this should incorporate relevant parameters of how the introduction of engineered *B*. *glabrata* would affect other parasite communities that parasitize these snails. Many of these parameters will need to be worked out through basic research of heterospecific interactions within individual snail species, as currently it is unclear how these interactions would affect persistence of resistant snails in the environment or how resistance to one parasite might affect susceptibility to another. Indeed, more needs to be known about the genetic determinants of *B*. *glabrata* resistance to other trematodes, especially because mechanisms of invasion and host immune interference can differ between digeneans parasitizing the same snail host [139]. Comparative immunology analyzing the host responses of *B*. *glabrata* to either schistosomes or echinostomes has provided an ideal platform for this work, and this model could aid predictions of how engineered snails may interact with the diverse number of parasites encountered in their environment. These interactions highlight the difficulty of translating an engineered *B*. *glabrata* strain to the field and predicting its widespread ecological impact, but the current knowledge gap is not impassable.

Basic research of genome editing of *B*. *glabrata* (initially including the Bge cell line) and other snail intermediate hosts should continue. In addition, because of the high global burden of schistosomiasis and the urgent need for transmission interruption, successful editing and transmission of an engineered allele should be immediately reported via preprint and subsequent publication, followed by well-contained laboratory and mesocosm trials. Even if the effectiveness of gene drives is supported by these preliminary investigations, its implementation and deployment will still face many obstacles. For example, the translation to field trials will be challenging. However, the necessary scientific and governance expertise is available in the community of schistosomiasis research and disease control, and the breadth of experiences within the malaria research community will provide an invaluable precedent and guide. Ultimately, through collaborative efforts and responsive scientific progress, a future scenario of schistosomiasis reduction or even elimination is possible.

## Supporting information

S1 Appendix Mathematical model used to illustrate the qualitative behavior of transmission patterns following successful introduction of engineered resistant snails. (DOCX)
