# Supplementary material for: Gene drives for schistosomiasis transmission control
Source: PLoS Negl Trop Dis. 2019 Dec 19;13(12):e0007833. doi: 10.1371/journal.pntd.0007833 (PMC6922350; doi:10.1371/journal.pntd.0007833)
Supplement: S1 Appendix — (DOCX) [file pntd.0007833.s001.docx]

**S1 File. Appendix**

**Modelling considerations**

Here we modify the classic MacDonald model for schistosome transmission to include the frequency of resistant snails that occurs in the environment due to introduction and subsequent spread of the gene drive construct in the population. The simplest form this takes is to subtract the frequency of resistant snails from the susceptible snail frequency such that the frequency of susceptible snails is given by $(1-y-\rho)$, where $\rho$ is the engineered resistant frequency. The resulting system of differential equations is given below.

$$\frac{dm}{dt}=\alpha Ny - \mu_{1}m$$

$$\frac{dy}{dt}=\beta Hm(1-y-\rho) - \mu_{2}y$$

$m$ and $y$ represent per capita mean worm burden and percent infected snails, respectively. $\alpha$ and $\beta$ are transmission parameters whose values are derived in the appendix. $N$ and $H$ give the size of the snails and human populations, respectively. $\mu_{1}$ is the per capita adult worm death rate, while $\mu_{2}$ is the per capita infected snail death rate. The distribution of adult worms in the human population is assumed to approximate a negative binomial distribution. All values and calculations are given in **Table 1**.

**Table 1:** Parameter and variable values derived from calculations and literature

| Parameter | Value | Description | Source |
| --- | --- | --- | --- |
| $\alpha$ | $1.0\underline{6} *10^{-3} \frac{worms}{infected snail*wk}$ | Per capita snail to human transmission rate | Calculated based on model equilibrium at $m^{*}=40, y^{*}=0.015$ |
| $N$ | $10^{4} snails$ | Size of snail population (assumed constant) | Approximation based on $50 \frac{snails}{m^{2}}$density [[1]](https://paperpile.com/c/yl6XBB/77J9) and water access size [[2](https://paperpile.com/c/yl6XBB/YMkt)] |
| $\mu_{1}$ | $0.004 wk^{-1}$ | Death rate of adult worms | Harmonic mean of range of $(3-7 yrs)^{-1}$commonly reported in literature across *Schistosoma spp.* [[3](https://paperpile.com/c/yl6XBB/gvRp)] |
| $\mu_{2}$ | $0.25 wk^{-1}$ | Death rate of infected snails | Harmonic mean of death rates of infected *Bulinus globosus* and *Biomphalaria pfeifferi* [[4](https://paperpile.com/c/yl6XBB/9okY)] |
| $b$ | $2.08*10^{-7}\frac{snails infected}{w*wk}$ | Per capita human to snail transmission rate | Calculated based on model equilibrium at $m^{*}=40, y^{*}=0.015$ |
| $w$ | Variable, $w^{*}=18.34$ | Per capita mean number of mated pairs of adult worms | Calculated: $w=\frac{1}{2}\phi m$ |
| $k$ | $0.24$ | Clumping parameter: $NB(m,k)$ | Fitted to *S. mansoni* data [[5](https://paperpile.com/c/yl6XBB/RV3E)] |
| $\beta$ | Variable, $\beta^{*}=9.52*10^{-8}\frac{snails infected}{humans*worms*wk}$ | Per capita worm to snail transmission rate | Calculated: $\beta=bw$ |
| $H$ | $10^{3} humans$ | Size of human population | Average size of rural African villages where *S. mansoni* and *S. haematobium* are endemic [[6](https://paperpile.com/c/yl6XBB/jDQd)] |
| $\rho$ | Variable, $[0,1]$ | Frequency of engineered snails resistant to infection | Varied continuously to approximate effect |
| $m$ | Variable, $m^{*}=40$ | Per capita mean worm burden | Calculated based on model equilibrium at $P=0.60$ |
| $y$ | Variable, $y^{*}=0.015$ | Frequency of patent infections in snail population | Field-observed average in many endemic regions [[7](https://paperpile.com/c/yl6XBB/fDVU)] |
| $\phi$ | Variable, $\phi^{*}=0.92$ | Per adult worm mating probability | Calculation based on $NB(m,k)$ |
| $\Omega_{t}$ | Variable, $\Omega_{t}^{*}=0.60$ | Per capita prevalence of at least one mated pair of adult worms | Field-observed average in many endemic regions [[8,9](https://paperpile.com/c/yl6XBB/FgFQ+JcJW)] |

This model does not include explicit evolutionary dynamics needed for full understanding of the introduction of gene-drive engineered snails. Instead, we rely on existing mathematical epidemiological framework to explore model behaviour for a set of fixed values for $\rho\in[0,1]$. Reduction in disease determinants commonly used to evaluate helminthiases can be measured, including prevalence of infection, intensity of infection as measured by egg output from mated worm pairs, and effective reproductive number, $R_{t}$, which is an epidemiological value for transmissibility. Simulations of 10 years of engineered snail intervention were run to determine reduction in disease burden as measured by these determinants. Fig 3 illustrates potential for disease reduction when 50% frequency of gene drive engineered snails can be maintained in a wild population from 0 to 10 years. Fig 3 also shows the magnitude of disease reduction that could occur under varying levels of intervention success. The frequency of gene drive engineered snails ($\rho$) varies from 0 to 1. Values are given as percent reduction from endemic equilibrium.

Setting $\rho=0$, we calculate transmission rates $\alpha$and $\beta$at endemic equilibrium. Let $m^{*}, y^{*}$be nontrivial equilibrium values for which $\frac{dm}{dt}=\frac{dy}{dt}=0$.

$m^{*}=\frac{\alpha Ny}{\mu_{1}}$, $y^{*}=\frac{\beta Hm}{\beta Hm+\mu_{2}}$

$y^{*}$is readily determined by available field data as the prevalence of patent infection in snails. $m^{*}$ is not as readily determined as estimates of mean worm burden in humans comes from the quantity of shed eggs in fecal or urine samples, and when prevalence of infection in humans is <<100%, a fraction of worms are unable to successfully reproduce due to failure to pair with a mate. A negative binomial distribution is found to best approximate the distribution of worms across humans in a local population. This distribution is overdispersed, and the probability of mating of any given worm is less than unity. Prevalence of detectable eggs, and thus successful mated pairs, in a human population at time $t$ is given by

$\Omega_{t}(m,k)=1-2(1+\frac{m}{2k})^{-k}+(1+\frac{m}{k})^{-k}$ [[10](https://paperpile.com/c/yl6XBB/DnTE)]

and $m$is calculated via substitution of known prevalence, $\Omega_{t}$, and estimated clumping parameter, $k$, and solving numerically. This calculation assumes equal ratio of male and female schistosomes transmitted, the rate of transmission of each sex is equal, both sexes are transmitted together, and there is no compartmentalization in the body that would limit pairing of adult schistosomes. $m^{*}=40$ occurs at an equilibrium prevalence, ${\Omega_{t}}^{*}$, of 60%. $\alpha$ can be calculated given $m^{*}=40, y^{*}=0.015$ as

$$\alpha=\frac{m^{*}\mu_{t}}{Ny^{*}}=1.0\underline{6}*10^{-3} worms(infected snail*wk)^{-1}$$

Although $\alpha$ is a constant parameter, $\beta$ is a function of the distribution of adult worms in the human population and changes non-linearly with prevalence and mean worm burden. A simple form $\beta$ can take is

$$\beta_{t}=\frac{1}{2}b\phi_{t}$$

where $b$ is a constant of transmission that relates the per capita number of worm pairs, $w$, to new snail infections.

$$w_{t}=\frac{1}{2}\phi_{t}m_{t}$$

$\phi$ describes the mating probability as determined by the given negative binomial distribution, where $\delta_{t}=\frac{m_{t}}{m_{t}+k}$.

$\phi_{t}=1-\frac{(1-\delta_{t})^{1+k}}{2\pi}\int_{0}^{2\pi} \frac{(1-cos\theta)d\theta}{(1+\delta_{t}cos\theta)^{1+k}}$ [[10](https://paperpile.com/c/yl6XBB/DnTE)]

Given $m^{*}$and $y^{*}$, $\beta^{*}$and $b$ are calculated as:

$$\beta^{*}=\frac{\mu_{2}y^{*}}{Hm^{*}(1-y^{*})}$$

$$b=\frac{2\beta^{*}}{\phi^{*}}$$

Table 1 summarizes these parameters and variables and gives values used to initialize simulations.

For compartmental epidemiological models it is standard practice to calculate $R_{0}$, the basic reproductive number for disease agents that describes the ability of a disease to persist. Usually this calculation is performed given a disease-free state to describe invasion conditions in the first generation for a fully susceptible host population and the introduction of the disease agent. $R_{0}>1$ is the criterion for successful establishment of the disease agent in the susceptible population. Because $\beta$ is a variable dependent on the worm burden in the human population, $R_{0}$ for this model is 0 beginning from a disease-free state. This can be shown by deriving the expression for $R_{0}$ assuming $\beta$ is a constant parameter.

$$R_{0}=\frac{\alpha N\beta H}{\mu_{1}\mu_{2}}$$

In a disease-free state, $w_{0}=\beta_{0}=R_{0}=0$. The positive feedback between $\beta$ and $m$ means $R_{t}$is a more appropriate descriptor of invasion dynamics for this model as 0 is an attractor below some critical threshold of disease [[11,12](https://paperpile.com/c/yl6XBB/eadD+z4pq)]. Nevertheless, $R_{t}$ can be used to evaluate persistence of a disease agent at time $t$ in an endemic system. In the case where engineered snails resistant to infection are added to a schistosoma-endemic area, the change in $R_{t}$ provides indication of the cumulative impact of intervention. This can be expressed as

$$R_{t}=\frac{\alpha N\beta_{t}H(1-\rho_{t})}{\mu_{1}\mu_{2}}$$

1. [Woolhouse MEJ, Chandiwana SK, Others. Population biology of the freshwater snail Bulinus globosus in the Zimbabwe highveld. J Appl Ecol. 1990;27(1):41–58.](http://paperpile.com/b/yl6XBB/77J9)

2. [Sokolow SH, Huttinger E, Jouanard N, Hsieh MH, Lafferty KD, Kuris AM, et al. Reduced transmission of human schistosomiasis after restoration of a native river prawn that preys on the snail intermediate host. Proc Natl Acad Sci U S A. 2015 Aug 4;112(31):9650–5.](http://paperpile.com/b/yl6XBB/YMkt)

3. [Goddard MJ, Jordan P. On the longevity of Schistosoma mansoni in man on St. Lucia, West Indies. Trans R Soc Trop Med Hyg. 1980;74(2):185–91.](http://paperpile.com/b/yl6XBB/gvRp)

4. [Woolhouse ME. The effect of schistosome infection on the mortality rates of Bulinus globosus and Biomphalaria pfeifferi. Ann Trop Med Parasitol. 1989 Apr;83(2):137–41.](http://paperpile.com/b/yl6XBB/9okY)

5. [Mangal TD, Paterson S, Fenton A. Predicting the impact of long-term temperature changes on the epidemiology and control of schistosomiasis: a mechanistic model. PLoS One. 2008 Jan 16;3(1):e1438.](http://paperpile.com/b/yl6XBB/RV3E)

6. [Webbe G, James C. Host-parasite Relationships of Bulinus globosus and B. truncatus with Strains of Schistosoma haematobiutm. J Helminthol. 1972 Jun;46(2):185–99.](http://paperpile.com/b/yl6XBB/jDQd)

7. [Anderson RM, May RM. Prevalence of schistosome infections within molluscan populations: observed patterns and theoretical predictions. Parasitology. 1979 Aug;79(1):63–94.](http://paperpile.com/b/yl6XBB/fDVU)

8. [Chan MS, Guyatt HL, Bundy DA, Booth M, Fulford AJ, Medley GF. The development of an age structured model for schistosomiasis transmission dynamics and control and its validation for Schistosoma mansoni. Epidemiol Infect. 1995 Oct;115(2):325–44.](http://paperpile.com/b/yl6XBB/FgFQ)

9. [Tchuem Tchuenté L-A, Momo SC, Stothard JR, Rollinson D. Efficacy of praziquantel and reinfection patterns in single and mixed infection foci for intestinal and urogenital schistosomiasis in Cameroon. Acta Trop. 2013 Nov;128(2):275–83.](http://paperpile.com/b/yl6XBB/JcJW)

10. [May RM. Togetherness among Schistosomes: its effects on the dynamics of the infection. Math Biosci. 1977 Jan 1;35(3):301–43.](http://paperpile.com/b/yl6XBB/DnTE)

11. [Klutse A, Baleux B. [Survival of Bulinus truncatus and Biomphalaria pfeifferi in sewer water purified in stabilization ponds in a sudanese-saharan zone]. Med Trop . 1996;56(1):41–7.](http://paperpile.com/b/yl6XBB/eadD)

12. [Gazzinelli MFC, Kloos H, de Cássia Marques R, dos Reis DC, Gazzinelli A. Popular beliefs about the infectivity of water among school children in two hyperendemic schistosomiasis areas of Brazil. Acta Trop. 2008 Nov 1;108(2):202–8.](http://paperpile.com/b/yl6XBB/z4pq)
